# Precision genome editing in plants via gene targeting and *piggyBac*-mediated marker excision

**DOI:** 10.1111/tpj.12693

**Published:** 2014-10-06

**Authors:** Ayako Nishizawa-Yokoi, Masaki Endo, Namie Ohtsuki, Hiroaki Saika, Seiichi Toki

**Affiliations:** 1 Plant Genome Engineering Research Unit, National Institute of Agrobiological Sciences2-1-2 Kannondai, Tsukuba, Ibaraki, 305-8602, Japan; 2 Kihara Institute for Biological Research, Yokohama City University641-12, Maioka-cho, Yokohama, 244-0813, Japan

**Keywords:** gene targeting, marker excision, *piggyBac* transposon, *acetolactate synthase*, *cleistogamy 1*, *Oryza sativa*, technical advance

## Abstract

Precise genome engineering via homologous recombination (HR)-mediated gene targeting (GT) has become an essential tool in molecular breeding as well as in basic plant science. As HR-mediated GT is an extremely rare event, positive–negative selection has been used extensively in flowering plants to isolate cells in which GT has occurred. In order to utilize GT as a methodology for precision mutagenesis, the positive selectable marker gene should be completely eliminated from the GT locus. Here, we introduce targeted point mutations conferring resistance to herbicide into the rice *acetolactate synthase* (*ALS*) gene via GT with subsequent marker excision by *piggyBac* transposition. Almost all regenerated plants expressing *piggyBac* transposase contained exclusively targeted point mutations without concomitant re-integration of the transposon, resulting in these progeny showing a herbicide bispyribac sodium (BS)-tolerant phenotype. This approach was also applied successfully to the editing of a microRNA targeting site in the rice *cleistogamy 1* gene. Therefore, our approach provides a general strategy for the targeted modification of endogenous genes in plants.

## Introduction

Gene targeting (GT) via HR between an endogenous gene and an exogenous targeting vector, and GT via mismatch repair with chimeric RNA/DNA oligonucleotide (chimeraplast) are established techniques that allow desired mutations to be introduced into target genes. Unless the inserted mutations confer a selectable trait, such as resistance to herbicides, HR-mediated GT with a positive–negative selection system is the current method of choice for selection of GT cells in several organisms. In flowering plants, HR-mediated GT with a positive–negative selection system has been developed as a universal approach and has been used successfully to modify several genes (Terada *et al*., 2002, 2007; Yamauchi *et al*., 2009; Moritoh *et al*., 2012; Ono *et al*., 2012; Dang *et al*., 2013). However, when using a positive–negative selection system, the positive selection marker gene inserted into the targeted locus should be removed completely from the GT locus if the intention is to introduce desired mutations exclusively.

Site-specific recombination systems such as the Cre/*loxP* and FLP/*FRT* system have been utilized widely to remove selectable marker genes from GT loci in mammals and plants (van der Weyden *et al*., 2002; Terada *et al*., 2010; Dang *et al*., 2013). However, site-specific recombination systems leave dispensable sequences, e.g., the 34-bp recognition sequences of site-specific recombinase (a single *loxP* and *FRT* site), at the excised site. Furthermore, such sequences have the potential to affect expression of adjacent genes in mammalian cells (Meier *et al*., 2010). In addition, it has been reported that the frequency of Cre/*loxP*-mediated marker elimination from a GT locus by transiently expressing Cre recombinase in rice was quite low (Terada *et al*., 2010; Dang *et al*., 2013).

In contrast, a system using a *piggyBac* transposon derived from the cabbage looper moth afforded precise genome modification via GT and subsequent marker excision in mammalian cells (Yusa *et al*., 2011a; Morioka *et al*., 2014; Sun and Zhao, 2014). In our previous study, we designed an assay system that allows *piggyBac* transposition to be visualized as luciferase luminescence in rice cells, and demonstrated that the *piggyBac* transposon is capable of accurate and effective transposase-mediated transposition also in plant cells (Nishizawa-Yokoi *et al*., 2014). In addition, we have demonstrated *piggyBac* transposition-mediated excision of a selectable marker from the reporter locus at high frequency without concomitant re-integration of the transposon (Nishizawa-Yokoi *et al*., 2014). Here, we applied *piggyBac*-mediated marker excision system to remove a positive selectable marker gene from a GT locus in rice.

## Results

### Introduction of point mutations into the *ALS* gene via GT using a positive–negative selection system

**Figure 1:**
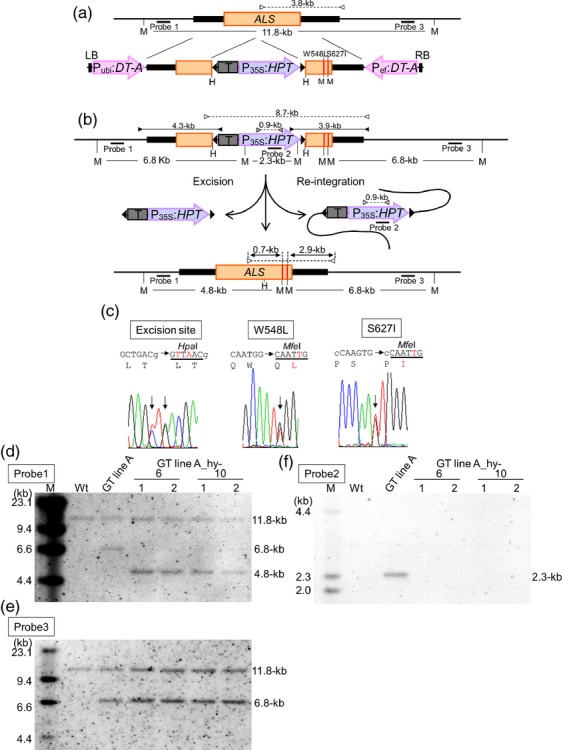
Strategy for the introduction of point mutations into the *ALS* locus via GT and subsequent marker excision from the GT locus using *piggyBac* transposon. (a) Schematic diagram of GT at the *ALS* locus. The top line indicates the genomic structure of the wild-type *ALS* gene region. The bottom line shows the T-DNA region of the targeting vector carrying *diphtheria toxin A subunit* gene (*DT-A*) under the control of the maize polyubiquitin 1 promoter (P_ubi_) or rice elongation factor-1α promoter (P_ef_) as negative selection marker and a 6.4-kb fragment containing an *ALS* coding region (open box) with W548L and S627I mutations (red lines) and silent mutations (added *Hpa*I site at 301-bp upstream of W548L; GCTGAC to GAATTC) for the insertion of *piggyBac* transposon (black triangle) harboring a rice actin terminator (T) and *hpt* gene under the control of the cauliflower mosaic virus 35S promoter (P_35S_) as positive selection marker. LB, left border; RB, right border. (b) Strategy for precise marker excision using *piggyBac* transposon from the GT locus. The top line reveals the structure of the modified *ALS* locus resulting from homologous recombination between the targeting vector and wild-type locus. The bottom line represents the *ALS* locus modified by GT and subsequent precise marker excision via *piggyBac* transposition. The primer sets used for PCR that identify transgenic calli in which a GT event occurred at *ALS* locus are shown as black arrows. White arrows indicate the primer sets used for CAPS analysis to evaluate the frequency of marker excision via *piggyBac* transposition. Gray arrows represent primers for PCR analysis to detect the existence of re-integrated *piggyBac* transposon. The numbers on each arrow reveal the length of the PCR fragments. (c) Sequencing chromatograms of the excision site and mutation site in T_0_ plants. (d–f) Southern blot analysis with probe1 (d), 2 (f), and 3 (e) shown in (a) and (b) using *Mfe*I-digested genomic DNA of wild-type, a regenerated plant of *ALS* GT-A and two T_0_ plants each of two independent lines with *ALS* GT-A_hy-6 and 10 (*ALS* GT-A_hy-6-1, 6-2, 10-1 and 10-2).

To date, we have successfully generated rice plants tolerant to the herbicide bispyribac sodium (BS) by introducing two point mutations specifying two amino acid changes – tryptophan (TGG) to leucine (TTG) at amino acid 548 (W548L), and serine (AGT) to isoleucine (ATT) at amino acid 627 (S627I) – in the *ALS* locus via GT, since the infrequent GT cells can be selected easily on medium containing BS (Endo *et al*., 2007). To establish a universal strategy for producing mutant plants harboring only the desired mutation in the target locus, we attempted to introduce the W548L and S627I mutations into the *ALS* gene locus via GT with positive–negative selection and subsequent excision of the positive selectable marker gene from the *ALS* gene locus using *piggyBac* transposon. In rice plants, a strong positive–negative selection system using the *hpt* gene conferring resistance to hygromycin B as positive selection marker and diphtheria toxin A subunit gene (*DT-A*) as a negative selection marker has been developed for the selection of GT-positive cells (Terada *et al*., 2002). The GT vector carries *DT-A* gene expression cassettes at both sides and a 6.4-kb fragment containing an *ALS* coding region with W548L and S627I mutations. The *piggyBac* transposon integrates into the host genome at TTAA elements and excises without leaving a footprint at the excised site (Cary *et al*., 1989). Therefore, *piggyBac* transposon harboring a rice actin terminator and *hpt* expression cassette was inserted at an *Hpa*I site with two additional silent mutations at the junctions to make an *Hpa*I site (gTTAAc) for *piggyBac* transposition (Figure 1a). The *ALS* gene modified with GT should be inactive since the *ALS* coding gene is interrupted by the *hpt* gene expression cassette flanked by sequences needed for *piggyBac*-mediated transposition.

Rice calli derived from mature seeds were inoculated with *Agrobacterium* harboring the GT vector and were selected on medium containing hygromycin B for 4 weeks (Figure S1). In total, 100 independent hygromycin-resistant calli were selected from 3259 (approximately 20 g) pieces of calli (Table 1) and were subjected to polymerase chain reaction (PCR) analysis with the primer sets shown in Figure 1(b) to identify transgenic calli in which GT events had occurred at the *ALS* locus. Both upstream and downstream junction fragments were detected in six independent selected callus lines, indicating that the positive selection marker gene was introduced into the *ALS* locus by homologous recombination (HR) between the GT vector and the target locus. Sequencing analysis revealed a lack of W548L or W548L/S627I mutations in the targeted *ALS* locus in two callus lines (*ALS* GT-B4 and B5), respectively. Four callus lines (*ALS* GT-A, B1, B2 and B3) were identified as GT calli (Table 1), and among them, two lines (*ALS* GT-A and B1) were used for the marker excision study.

**Table 1:** Summary of GT experiments targeting the *ALS* locus

Experiments	No. of *Agrobacterium* infected calli	No. of hygromycin- resistant calli	No. of targeted calli	No. of targeted calli with W548L/S627I mutations
A	1463 (7.53 g)	25	1	1
B	1796 (11.85 g)	75	5	3
Total	3259 (19.38 g)	100	6	4

### Precise marker excision from the GT locus by *piggyBac* transposition

*ALS* GT-A and B1 calli were infected with *Agrobacterium* harboring a hyperactive *piggyBac* transposase (hyPBase) (Yusa *et al*., 2011a, b) expression vector driven by the constitutive maize polyubiquitin gene 1 (Ubi-1) promoter (designated *ALS* GT-A_hy and B1_hy, Figure S1). Transgenic calli were selected on N6D medium with geneticin and meropenem, and transferred to regeneration medium with meropenem. Batches of twenty T_0_ regenerated plants from five or six independent *ALS* GT-A_hy and B1_hy lines, respectively, were subjected to marker excision analysis by cleaved amplified polymorphic sequences (CAPS) that combined PCR amplification and restriction digestion with *Mfe*I as described in Experimental Procedures.

The two point mutations in the targeted *ALS* locus generate recognition sites for the restriction enzyme *Mfe*I (CAATTG). Therefore, *Mfe*I digestion of PCR fragments derived from the wild-type *ALS* locus or GT-modified *ALS* locus and subsequent marker excision is expected to generate a non-digested 3.8-kb fragment or fragments of 2.9-, 0.7- and 0.2-kb, respectively (Figure 1a,b). CAPS analysis with genomic DNA extracted from leaves of *ALS* GT-A_hy and B1_hy revealed the expected *Mfe*I-digested fragment in candidate marker-free rice plants containing W548L/S627I mutations in the *ALS* locus. More than 90% of regenerated plants (on average, 100 and 92.5% of *ALS* GT-A_hy and B1_hy, respectively; Tables 2 and S1) contained *Mfe*I-digested fragments, indicating that the *piggyBac* has transposed effectively from the targeted *ALS* locus via hyPBase. Furthermore, whether the excised *piggyBac* transposon is re-integrated into other loci was analyzed by PCR using primers specific to the *hpt* coding region generating a 0.9-kb PCR fragment (Figure 1b). Only one plant of each *ALS* GT-A_hy and B1_hy regenerated plants harboring the *Mfe*I-digested fragment by CAPS analysis contained *hpt*-specific fragments (Tables 2 and S1). Thus, 99 and 92% of *ALS* GT-A_hy and B1_hy regenerated plants, respectively, were found to be marker-free rice plants containing two point mutations W548L and S627I in the *ALS* locus. Direct sequencing of PCR fragments from eight and 16 randomly selected plants of *ALS* GT-A_hy and B1_hy revealed the presence of W548L/S627I mutations and silent mutations (added *Hpa*I site) for insertion of the *piggyBac* transposon in all regenerated plants analyzed (Figure 1c). Therefore, our findings indicate that mutant plants containing desired point mutations and lacking residual sequences in the target locus were obtained successfully in a convenient manner.

**Table 2:** PCR analysis of *piggyBac* excision and re-integration events in *ALS* GT-A_hy regenerated plants by hyPBase expression

		*piggyBac* excision from *OsALS* locus			Frequency of *piggyBac* excision (%)		
Line no.	No. of T_0_ plants analyzed	Without marker	With marker	Total	Without re-integration	With re-integration	Total
5	20	19	1	20	95.0	5.0	100
6	20	20	0	20	100	0	100
10	20	20	0	20	100	0	100
13	20	20	0	20	100	0	100
24	20	20	0	20	100	0	100
Average					99.0	1.0	100

To confirm the introduction of W548L/S627I mutations via GT and the excision of the selectable marker gene by *piggyBac* excision in the *ALS* locus in T_0_ plants, we performed Southern blot analysis with genomic DNA extracted from wild-type, a regenerated plant of *ALS* GT-A and two T_0_ plants each of two independent lines with *ALS* GT-A_hy-6 and 10. Southern blot analysis of *Mfe*I-digested DNA with probe1 (Figure 1d) showed that wild-type bands (11.8-kb) and bands indicating gene targeting (6.8 kb) were detected in *ALS* GT-A regenerated plants (Figure 1d), while wild-type bands (11.8 kb) and 4.8-kb bands derived from *piggyBac*-excised targeted *ALS* locus were detected using probe1 in *ALS* GT-A_hy regenerated plants (Figure 1d). In addition to the wild-type band (11.8-kb), 6.8-kb bands derived from the *ALS* locus containing W548L/S627I mutations via GT were observed using probe3 in *ALS* GT-A and GT-A_hy regenerated plants (Figure 1e). Furthermore, Southern blot analysis with an *hpt* probe revealed 2.3-kb bands derived from a positive selection marker only in *ALS* GT-A, indicating that excised *piggyBac* had not re-integrated into other loci in *ALS* GT-A_hy regenerated plants (Figure 1f). Consistent with these results, we confirmed the introduction of W548L/S627I mutations without any *piggyBac* footprint in the *ALS* locus in T_0_ plants of *ALS* GT-B1_hy-19 and 20 by Southern blot analysis (Figure S2a–c).

To further verify our GT-mediated precision mutagenesis system, a single base substitution (CAGCAGCA/GTCATCACGATTCC) was introduced by GT into the microRNA targeting site of the rice *cleistogamy 1* (*Oscly1*) gene (Chen, 2004; Nair *et al*., 2010) – an orthologue of *Arabidopsis thaliana* AP2 transcription factor involved in the specification of floral organ identity (Jofuku *et al*., 1994) – and the selectable marker was subsequently excised from the GT locus using *piggyBac* transposon (Figure S3a,b). Four independent callus lines were identified as GT calli from 5139 (approximately 25.6 g) pieces of calli by PCR analysis with the primer sets shown in Figure S3(b) and subsequent sequence analysis (Table S2). Two independent lines (*cly1* GT-1 and -2) were infected with *Agrobacterium* harboring a hyPBase expression vector (designated *cly1* GT-1_hy and GT-2_hy) and were subjected to marker excision analysis. Via PCR with the primer sets shown in Figure S3(b) and sequence analysis, we confirmed that 91% and 98% of *cly1* GT-1_hy and GT-2_hy regenerated plants, respectively, contained the desired A to G mutation in the microRNA targeting site of the *Oscly1* gene but not the positive selectable marker gene (Figure S3a–c and Tables S3 and S4). Furthermore, Southern blot analysis revealed that all the regenerated plants so far analyzed were marker-free plants, and that there was no re-integration of *piggyBac* transposon (Figures S3d–f). Taken together, these results validated accurate and effective *piggyBac*-mediated marker excision from the *Oscly1* locus. Detailed characterization of *Oscly1*-mutated rice will be presented elsewhere.

### Analysis of marker-free T_1_ progeny harboring an *ALS* gene modified via GT

**Figure 2:**
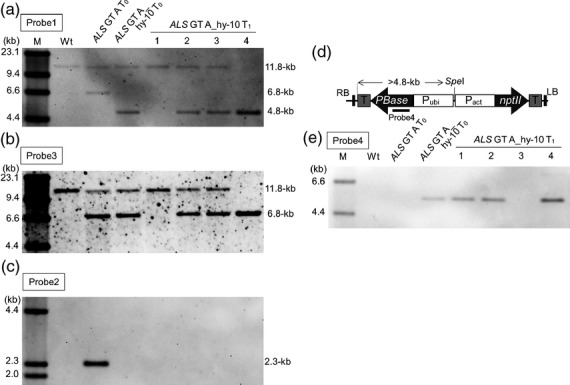
Segregation of the *ALS* gene harboring W548L/S627I mutations and hyPBase expression vector in T_1_ progeny. (a–c) Southern blot analysis with probe 1 (a), 2 (c), 3 (b) shown in Figure 1(a,b) using *Mfe*I-digested genomic DNA of wild-type, a regenerated plant of *ALS* GT-A and *ALS* GT-A_hy-10, and four T_1_ progeny of ALS GT-A_hy-10 (ALS GT-A_hy-10-1, 2, 3, and 4). Southern blot analysis revealed that the *ALS* locus with W548L/S627I segregated in T_1_ progenies of ALS GT-A_hy-10 (wild-type *ALS* gene, line no. 1; heterozygous modification of the *ALS* gene, line no. 2 and 3; homozygous modification of *ALS* gene, line no. 4). (d) Schematic diagram of hyPBase expression vector. The hyPBase expression vector carries a rice Ubi-1 promoter (Pubi)::hyPBase (*PBase*) cassette and a rice actin promoter (Pact)::*nptII* expression cassette. T, terminator; LB, left border; RB, right border. (e) Southern blot analysis with probe 4 shown in (d) using *Spe*I-digested genomic DNA of wild-type, a regenerated plant of *ALS* GT-A and *ALS* GT-A_hy-10, and four T_1_ progeny of ALS GT-A_hy-10 (ALS GT-A_hy-10-1, 2, 3, and 4). The hyPBase expression vector segregated out in *ALS* GT-A_hy 10-3.

T_1_ progeny plants were obtained from self-pollinating *ALS* GT-A_hy-10 T_0_ plants. Genomic DNA was extracted from four progenies of *ALS* GT-A_hy-10 T_0_ regenerated plants and subjected to Southern blot analysis. Analysis of *Mfe*I-digested DNA with probe1 or probe3 (Figure 1b) showed that a plant with wild-type *ALS* locus (*ALS* GT-A_hy 10-1), heterozygous plants (*ALS* GT-A_hy 10-2, -3), and a homozygous (*ALS* GT-A_hy 10-4) plant with the modified *ALS* locus were obtained (Figure 2a,b). All of these T_1_ progeny lacked the *hpt*-specific fragment, indicating that the *piggyBac* transposon with the selectable marker gene had transposed and excised from the *ALS* locus without re-integration (Figure 2c). Furthermore, a *hyPBase*-specific fragment segregated out and was not detected in *ALS* GT-A_hy 10-3 calli by Southern blot analysis (Figure 2d,e).

**Figure 3:**
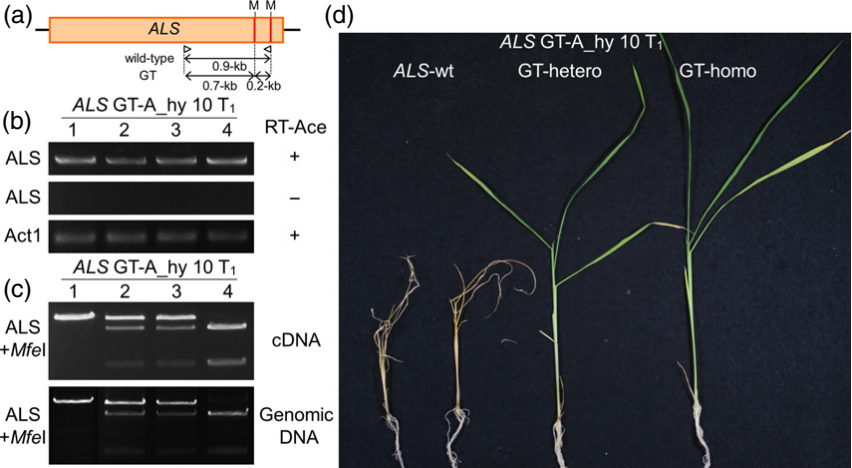
Analysis of the *ALS* gene harboring W548L/S627I mutations in T_1_ progeny. (a) Diagram showing the targeted ALS locus. Arrowheads indicate the primers used for the reverse transcriptase-polymerase chain reaction (RT-PCR, b) and CAPS (c). The expected band sizes of the RT-PCR (0.9-kb) and CAPS (wild-type, 0.9-kb; GT 0.7-kb and 0.2-kb) are shown. (b) Transcript levels of the *ALS* gene in T_1_ plants carrying the wild-type (line no. 1) and modified *ALS* gene (heterozygous, lines no. 2, 3 or homozygous, line no. 4). Top and middle panels show RT-PCR analysis using the *ALS* gene-specific primers with (top) or without (middle) reverse transcriptase (RT-Ace + or −, respectively). The *Actin 1* (*Act1*) gene was used as an internal control (bottom panel). (c) CAPS analysis combining PCR analysis using *ALS* gene-specific primers with cDNA (top) or genomic DNA (bottom) and *Mfe*I digestion in T_1_ plants carrying the wild-type (line no. 1) or modified *ALS* gene (heterozygous, lines no. 2, 3 or homozygous, line no. 4). (d) Herbicide bispyribac (BS)-tolerant phenotype of T_1_ plants. GT line A_hy T_1_ plants carrying the modified *ALS* gene [either heterozygous (GT-hetero) or homozygous (GT-homo)] showed BS tolerance after 3 weeks of BS treatment, but not T_1_ plants carrying the wild-type *ALS* gene (*ALS*-wt).

To examine whether the introduction of W548L and S627I mutations into the *ALS* gene via GT confers herbicide BS-tolerant phenotype in these T_1_ progeny plants, *ALS* gene transcript levels in rice calli derived from *ALS* GT-A_hy 10-1 (wild-type *ALS* locus, *ALS*-wt), 10-2, 10-3 (heterozygous plants, GT-hetero), and 10-4 (homozygous with the modified *ALS* locus, GT-homo) lines were analyzed by reverse transcription-polymerase chain reaction (RT-PCR) and BS-susceptibility test. We confirmed that there was no change in *ALS* gene transcription levels between *ALS*-wt, GT-hetero and GT-homo calli (Figure 3a–c). After 3-weeks of culture on callus induction medium containing 0.75 μm BS, GT-hetero and GT-homo calli with the modified *ALS* gene showed BS tolerance (Figure S4). Furthermore, the acquisition of BS tolerance was confirmed in segregated T_1_ progeny plants (Figure 3d).

## Discussion

The efficiency of positive–negative selection with the *hpt* and *DT-A* genes (GT-positive calli per hygromycin-resistant calli: 4.0%, Table 1) in this study was comparable to previous results (0.053–5.3%) (Terada *et al*., 2002, 2007; Yamauchi *et al*., 2009; Moritoh *et al*., 2012; Ono *et al*., 2012; Dang *et al*., 2013). Our previous report showed that approximately 1500 rice calli were transformed by *Agrobacterium* harboring a GT vector carrying the 5′-truncated *ALS* coding region with two mutations (W548L/S627I) and the downstream region of the *ALS* gene – namely a target gene-dependent selection – and 66 independent GT plants were obtained by selection on medium containing BS (Endo *et al*., 2007). Differences in the GT frequency between the previous study and this present work (GT-positive calli per callus infected by *Agrobacterium*: 4/3259) might be attributed to the integration of multiple T-DNA copies into the host genome, since random integration of the GT vector concomitant with sequence-specific integration via HR kills GT cells due to expression of the *DT-A* gene. In fact, extra bands in addition to the true GT fragment were detected by Southern blot analysis in GT lines obtained without negative selection (Endo *et al*., 2007). Saika and Toki (2010) reported that single copy T-DNA integration was observed to have occurred in <40% of isolated secondary calli of Nipponbare (on average, 2.6 T-DNA copies per transgenic callus). In addition, DT-A proteins directly cause lethality in cells, suggesting that transient expression of DT-A might be one of the causes of loss of GT frequency.

Using a GT procedure with positive–negative selection, an entire *hpt* expression cassette should be inserted into the *ALS* locus by HR regardless of the presence or absence of the two point mutations. In circumstances where GT occurs in somatic plant cells, DNA double-strand breaks (DSBs) are thought to be repaired mainly by synthesis-dependent strand annealing (SDSA) using the targeting vector as a repair template (Puchta and Fauser, 2013). In SDSA, single-strand overhangs with the 3′ end of the DSB site invade the repair template to form a D-loop structure and copy genetic information. Subsequently, 3′ ends are detached from the D-loop structure and re-anneal with the second strand at the DSB site. According to the SDSA model, the exogenous DNA information – *hpt* expression cassette – on the repair template is thought to inhibit homology search and homologous pairing between the 3′ end of the DSB site in the *ALS* locus and the GT vector at the step of strand invasion. In addition, the existence of a large exogenous DNA stretch within sequences homologous to the *ALS* locus may lead to a one-sided invasion, in which one end of the DSB is repaired by HR and the other end is rejoined by non-homologous end-joining (NHEJ). Because DNA synthesis is halted to allow copying of the repair template and resolution of the D-loop structure on *hpt* expression cassette is assumed to occur, the 3′ end of the DSB cannot re-anneal with the second strand at the DSB site. Thus, the extent of exogenous DNA information that needs to be copied/pasted into the target locus using positive–negative selection is larger than that needed in target gene-dependent selection, resulting in a decreased frequency of GT with positive–negative selection.

Consistent with previous reports (Endo *et al*., 2007; Johzuka-Hisatomi *et al*., 2008; Saika *et al*., 2011), the lack of either or both of the desired W548L/S627I mutations in the *ALS* gene were found in two callus lines out of six independently selected callus lines harboring the positive selection marker in the *ALS* locus (Table 1). Therefore, our findings could be explained by the occurrence of mismatch correction of the heteroduplex molecules formed between the genomic DNA and targeting vector or resolution of D-loop structures between the positive selection marker cassette and the W548L mutation or W548L and S627I mutations.

About 30% of *piggyBac* excision events from GT loci were reported to be accompanied by re-integration of *piggyBac* in mammalian cells under negative selection (Yusa *et al*., 2011a, b). In contrast, re-integration of excised *piggyBac* from the targeted *ALS* locus was observed in only 1% of calli analyzed (Tables 2 and S1). Our results might derive from expression of hyPBase protein from the stably integrated *hyPBase* gene under a strong promoter, i.e. re-integrated *piggyBac* transposon could be transferred again before finally being deleted from the plant chromosome. While, excision of a selection marker via hyPBase-induced *piggyBac* transposition from a reporter locus was detected previously in 72 and 91% of cases in two independent lines of transgenic T_0_ plants (Nishizawa-Yokoi *et al*., 2014). In this report, the frequency of excision of the *piggyBac* transposon from the GT locus was consistently over 90% (Tables 2, S1, S3 and S4). Our data raise the possibility that *piggyBac* can transpose easily from the GT locus regardless of the location of *piggyBac* insertion in the genome. This hypothesis can be explained by the findings of two previous reports: (i) *piggyBac* transposition has been reported to be inhibited by DNA methylation in mouse cells (Wang *et al*., 2008); and (ii) the DNA methylation profile of genomic loci modified by GT is altered in Arabidopsis (Lieberman-Lazarovich *et al*., 2013).

Recent developments in sequence-specific nucleases (SSNs) such as zinc finger nucleases (ZFNs), transcription activator-like effector nucleases (TALENs) and the bacterial clustered regularly interspaced short palindromic repeats (CRISPR)/Cas system, have enabled targeted DNA modification of plant genomes (Chen and Gao, 2013; Gaj *et al*., 2013; Voytas, 2013; Kim and Kim, 2014). DSBs induced by SSNs are rejoined by the NHEJ pathway, which can occasionally introduce mutations at the DSB sites. NHEJ-induced mutations are largely short deletions, but large deletions, insertions and nucleotide substitutions also occur infrequently (Chen and Gao, 2013; Gaj *et al*., 2013; Voytas, 2013; Kim and Kim, 2014). Therefore, it is difficult to introduce mutations designed to modify the properties of a target gene by SSNs-induced DSBs.

Because GT frequency in higher eukaryotes can be enhanced by DSBs created by SSNs (Puchta and Fauser, 2013), a negative selectable marker may not be absolutely necessary for the generation of GT plants. However, a positive selectable marker is required for selection of transformed cells from a large proportion of non-transformed cells. Therefore, a precise system of marker excision from GT loci using *piggyBac* transposon will become an indispensable technology for targeted gene modification leaving no residual ectopic sequences in the plant genome.

## Experimental procedures

### Vector construction

The GT vector was constructed as follows. The binary vector pKOD4 was constructed using the vector pKO3 (Osakabe *et al*., 2014) with two cytosine deaminase (*codA*) gene expression cassettes. A 0.6-kb diphtheria toxin (DT-A) (Terada *et al*., 2002) fragment was replaced by *codA* genes in the pKO3 vector, yielding pKOD4 with two *DT-A* gene expression cassettes (maize polyubiquitin 1 promoter + DT-A + rice heat shock protein (hsp) 16.9a terminator and rice elongation factor-1α promoter + DT-A + rice hsp 16.9b terminator). A 6.4-kb fragment containing the *ALS* gene carrying two point mutations of W548L and S627I (Endo *et al*., 2007) was digested with *Hpa*I/*Eco*RI and integrated into the *Sna*BI/*Eco*RI site of pENTR L1/L2 (Life Technologies, http://www.lifetechnologies.com). The *piggyBac* inverted-repeat transposable element (IVR) harboring meganuclease I*–Sce*I site (Nishizawa-Yokoi *et al*., 2014) was cloned into a *Hpa*I site (silent mutations at 301-bp upstream of W548L; GCTGAC to GAATTC) introduced by Expression-PCR (Lanar and Kain, 1994) using the primers 5′-tgctggatgagttaacgaaaggtgagg-3′ and 5′-cctcacctttcgttaactcatccagca-3′ in the *ALS* gene, yielding pE(L1-L2)mALSpb.

A 6.0-kb fragment containing the *Oscly1* gene was amplified by PCR from rice genomic DNA (Nipponbare) using the primers 5′-tt*ggcgcgcc*ttgtcgtcacgcgccagttc-3′ (*Asc*I site in italics) and 5′-cc*ttaattaa*tccagggaaatccaccactactact-3′ (*Pac*I site in italics) and integrated into the *Asc*I/*Pac*I site of pENTR L1/L2 (Life Technologies), yielding pE(L1-L2)*Oscly1*. A 429-bp artificially synthesized fragment containing the 10th exon and 3′-UTR of *Oscly1* harboring a single base substitution (CAGCAGCATCATCACGATTCC to CAGCAGCGTCATCACGATTCC in the putative microRNA target site at 88-bp upstream of the stop codon) carrying the *piggyBac* IVR in the TTAA site of the *Oscly1* 3′-UTR was replaced by the wild-type *Oscly1* gene in pE(L1-L2)*Oscly1*, yielding pE(L1-L2)*Oscly1*pb.

A 4.3 kb fragment containing the rice actin terminator, cauliflower mosaic virus 35S promoter, hygromycin phosphotransferase gene (*HPT*) and rice heat shock protein 17.3 terminator was digested with I*–Sce*I and integrated into pE(L1-L2)mALSpb or pE(L1-L2)*Oscly1*pb, yielding pE(L1-L2)mALSpHPTb and pE(L1-L2)Oscly1pHPTb, respectively. The fragment of mALSpHPTb or Oscly1pHPTb was re-cloned into the gene targeting binary vector pKOD4 using a Gateway LR clonase II reaction (Life Technologies), yielding pKOD4/mALS and pKOD4/Oscly1pHPTb, respectively. pKOD4/mALS or pKOD4/Oscly1pHPTb vector was transferred into *Agrobacterium tumefaciens* strain EHA105 (Hood *et al*., 1993) by electroporation.

### Plant materials and *Agrobacterium*-mediated transformation

*Agrobacterium*-mediated transformation of rice was performed as described previously (Toki *et al*., 2006). Rice (Nipponbare) was used for GT transformation following a method described previously (Saika *et al*., 2011). Calli transformed with *Agrobacterium* harboring pKOD4/mALS were selected on callus induction (N6D) medium solidified with 0.4% gelrite (Wako Pure Chemical Industries) containing 50 mg/L hygromycin and 25 mg/L meropenem (Wako Pure Chemical Industries, http://www.wako-chem.co.jp/). GT candidate calli were transferred to N6D medium without hygromycin and meropenem and cultured for 1 month. For marker excision, GT candidate calli were transformed with *Agrobacterium* to introduce an expression vector encoding hyPBase, and were selected on N6D medium with 35 mg/L geneticin (Nacalai tesque, https://www.nacalai.co.jp) and 25 mg/L meropenem. For regeneration, transgenic calli were transferred to regeneration medium with 25 mg/L meropenem, and shoots arising from callus were transferred to Murashige and Skoog (MS) medium (Murashige and Skoog, 1962) without phytohormones. The hyPBase-expressing regenerated plants were subjected to marker excision analysis. The T_1_ progeny plants were obtained from self-pollinating marker-free T_0_ plants containing two point mutations, W548L and S627I, in the *ALS* locus and were subjected to further analysis.

### Screening of GT candidate by PCR

After a 4-week selection period, hygromycin-resistant calli were subjected to screening by PCR. Genomic DNA was extracted from small pieces of rice calli using Agencourt chloropure (Beckman Coulter, https://www.beckmancoulter.com) according to the manufacturer's protocol. PCR amplifications were performed with KOD FX or KOD FX neo (TOYOBO) using the primer sets as follows: for 5′ amplification of ALS, ALS GT-F (5′-gacatgacaaccagtcatccgattaggttt-3′) and Tact-R (5′-ctgacgatgagaatatatctgatgctgtga-3′); for 3′ amplification of ALS, Thsp17.3-F (5′-acatacccatccaacaatgttcaatccctt-3′) and ALS GT-R (5′-tctggagatagcatacttgctttgcttggt-3′); for 5′ amplification of *Oscly1*, *Oscly1* GT-F (5′-tcggtcggctaaggtttgctactaaaaaca-3′) and Tact-R (5′-ctgacgatgagaatatatctgatgctgtga-3′); for 3′ amplification of Oscly1, Thsp17.3-F (5′-acatacccatccaacaatgttcaatccctt-3′) and *Oscly1* GT-R (5′-cttgcacgacggttctacaggagattagtg-3′).

### Sequence analysis

A 4302-bp or 3943-bp fragment was amplified using primer sets ALS GT-F/Tact-R and Thsp17.3-F/ALS GT-R from *ALS* GT-A or -B, respectively. A 3942-bp or 4257-bp fragment was amplified using primer sets *Oscly1* GT-F/Tact-R and Thsp17.3-F/*Oscly1* GT-R from *Oscly1* GT, respectively. These fragments were cloned into the vector pCR-Blunt II-TOPO using TOPO cloning methods (Life Technologies) and sequenced using universal primers M13-R (5′-caggaaacagctatgac-3′) and M13-F (5′-gtaaaacgacggccagt-3′) to confirm whether the junction sequence corresponded to the anticipated junction fragment. Primers ALS-R1 (5′-acttgggatcataggcagca-3′) and ALS-R2 (5′-ccttagcagtcaggaatagcttg-3′) were used to check the presence of the W548L and S627I mutations on a fragment amplified by Thsp17.3-F/ALS GT-R primers. Primer *Oscly1* Seq-5101F (5′-cgaccagaactcgaaccatc-3′) was used to check the presence of the single base substitution in the putative microRNA target site on a fragment amplified by *Oscly1* GT-F/Tact-R.

### *piggyBac* excision analysis by PCR

To evaluate the frequency of marker excision via *piggyBac* transposition from *ALS* GT locus, we performed CAPS analysis, which is PCR analysis coupled with *Mfe*I digestion (Endo *et al*., 2007). A 3.8-kb *piggyBac*-excised fragment was amplified with PrimeSTAR GXL DNA polymerase (TaKaRa, http://www.clontech.com/takara) using primers ALS-F1 (5′-gtacgcaaattatgccgtgga-3′), which anneals to the 359 bp upstream of the insertion site of *piggyBac*, and ALS GT-R, which is specific for the endogenous *ALS* locus (3478 bp downstream of the insertion site of *piggyBac* (Figure 1a). If *piggyBac* transposon is eliminated from the targeted *ALS* locus by hyPBase expression, PCR analysis and subsequent *Mfe*I digestion generates fragments of 2.9-, 0.7- and 0.2-kb (Figure 1a,b). In addition to these fragments, 3.8-kb fragments that could not be digested by *Mfe*I were detected in T_0_ regenerated plants owing to its heterozygosity. However, if *piggyBac* transposon remains at the targeted *ALS* locus in hyPBase-expressing regenerated plants, the expected amplified fragments including the selectable marker gene sequence should be 8.7-kb, which cannot be amplified with the PCR conditions used here (Figure 1b). To assess whether the selectable marker was excised precisely from the *Oscly1* GT locus via *piggyBac* transposition, we performed PCR analysis using primer sets *Oscly1* GT-F/Tact-R and *Oscly1* 3′ UTR-F (5′-ggatgctattcttttgctctaccttttt -3′), which anneals 380-bp upstream of the insertion site of *piggyBac*, and *Oscly1* 3′ UTR-R (5′-ttactttagtaccaacatctagaaggacga-3′), which anneals 223-bp downstream of the insertion site of *piggyBac*. If the *piggyBac* transposon is eliminated from the targeted *Oscly1* locus by hyPBase expression, a 0.6-kb fragment is amplified using the primer sets *Oscly1* 3′ UTR-F/-R; however, a 5.5-kb and 3.9-kb fragment including the selectable marker is not amplified using the primer sets *Oscly1* 3′ UTR-F/-R and *Oscly1* GT-F/Tact-R, respectively. To analyze the re-integration frequency of *piggyBac*, PCR analysis was performed with PrimeSTAR GXL DNA polymerase (TaKaRa) using primers HPT-F (5′-caaagatcgttatgtttatcggcactttg-3′) and HPT-R (5′-ctcgagctatttctttgccctc-3′) (Figures 1b and S3).

### Southern blot analysis

Genomic DNA was extracted from leaves of seedlings using the Nucleon Phytopure extraction kit (GE Healthcare, http://www3.gehealthcare.com/) according to the manufacturer's protocol. Two μg genomic DNA was digested with *Mfe*I and fractionated in a 1.0% agarose gel. Southern blot analysis was performed according to the digoxigenin (DIG) Application Manual (Roche Diagnostics, http://www.roche.com/). Specific DNA probes for the *ALS* and *Oscly1* locus were synthesized with a PCR DIG probe synthesis kit (Roche Diagnostics) according to the manufacturer's protocol, using the primers ALS probe-1 (5′-ttctttttcaatactttcctcgcttgctct-3′ and 5′-attcagccacttatcttgacacaaccattt-3′); and ALS probe-3 (5′-caaagatcgttatgtttatcggcactttg-3′ and 5′-ctcgagctatttctttgccctc-3′); *Oscly1* probe-1 (5′-ggttccattccctgacccggcccacct-3′ and 5′-cagtgaatgatgcaacatgagaccgaaca-3′); *Oscly1* probe-3 (5′-cagtcatctggacttgttggaattg-3′ and 5′-catcggatgagaccacattaactt-3′); probe-2 (5′-tgtgacagcccagtcatcat-3′ and 5′-cgttggatcgacatcatcag-3′); probe-4 (5′-atcagatgcctgaggatggac-3′ and 5′-acctgctgttcttcaggacctc-3′).

### RNA extraction and reverse transcriptase-polymerase chain reaction analysis

Total RNA was extracted from rice calli using an RNeasy Plant Mini Kit (Qiagen, http://www.qiagen.com/). First-strand cDNA was synthesized using ReverTra Ace (Toyobo, http://www.toyobo-global.com/) with oligo(dT_20_) primer. The cDNA encoding *ALS* was amplified from the first-strand cDNA by PCR using specific primers, 5′-gtacgcaaattatgccgtgag-3′ and 5′-acttgggatcataggcagca-3.’ To distinguish between cDNA from wild-type *ALS* gene and modified *ALS* gene, the first-strand cDNA was digested with *Mfe*I.

### BS-susceptibility test

Four-week-old rice calli derived from *ALS* GT-A_hy 10 T_1_ were transferred to callus induction medium containing 0.75 μm BS and cultured in a growth chamber under normal conditions. Three-week-old *ALS* GT-A_hy 10 T_1_ plants were transferred to ½MS medium (Murashige and Skoog, 1962) containing 1.5 μm BS and grown in a growth chamber at 29°C under continuous light. Photographs were taken 3 weeks after BS treatment in both cases.

## SUPPORTING INFORMATION

**Figure S1.** Experimental strategy for precise genome modification using the *piggyBac* transposon.

**Figure S2.** Molecular analysis of ALS GT-B1 and ALS GT-B1_hy T_0_ plants.

**Figure S3.** Strategy for the introduction of point mutations into *Oscly1* locus via GT and subsequent marker excision from the GT locus using *piggyBac* transposon.

**Figure S4.** Herbicide bispyribac (BS)-tolerant phenotype of T_1_ calli.

**Table S1.** PCR analysis of *piggyBac* excision and re-integration events in *ALS* GT-B1_hy regenerated plants by hyPBase expression

**Table S2.** Summary of GT experiments targeting the *Oscly1* locus.

**Table S3.** PCR analysis of *piggyBac* excision and re-integration events in *cly1* GT-1_hy plants regenerated by hyPBase expression

**Table S4.** PCR analysis of *piggyBac* excision and re-integration events in *cly1* GT-2_hy regenerated plants by hyPBase expression
