# Supplementary material for: Precision genome editing in plants via gene targeting and piggyBac-mediated marker excision
Source: Plant J. 2014 Oct 6;81(1):160–8. doi: 10.1111/tpj.12693 (PMC4309413; doi:10.1111/tpj.12693)
Supplement: Supplementary file 1 — Figure S1. Experimental strategy for precise genome modification using the piggyBac transposon. [file tpj0081-0160-sd1.docx]

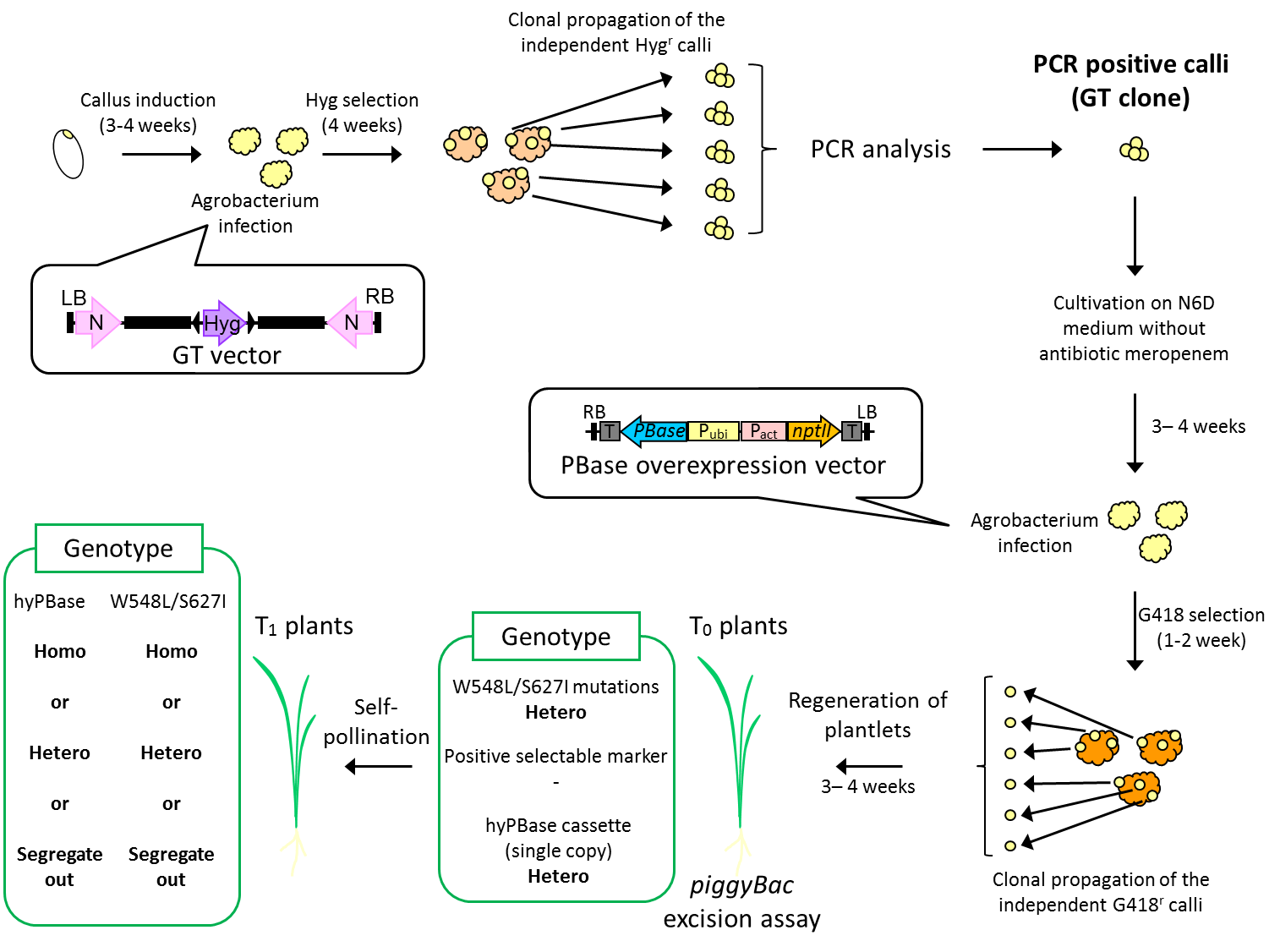


**Figure S1** Experimental strategy for precise genome modification using the *piggyBac* transposon.

Four-week-old rice calli were inoculated with *Agrobacterium* harboring GT vector pKOD4/mALS and were selected for 4 weeks on medium containing 50 mg/l hygromycin B. Genomic DNA extracted from hygromycin-resistant calli was subjected to PCR analysis with the primer sets shown in **Figure 1b** to identify transgenic calli in which GT events had occurred at the *ALS* locus. GT callus lines were transferred to medium without the antibiotic meropenem, which kills *Agrobacterium*, and were cultured for 4 weeks. GT calli were again infected with *Agrobacterium* harboring an pPN/hyPBase expression vector (Nishizawa-Yokoi et al., 2014) encoding hyperactive *piggyBac* transposase (hyPBase) (Yusa et al., 2011) driven by the maize poly-ubiquitin gene 1 (Ubi-1) promoter. hyPBase transgenic calli were selected on medium containing geneticin (G418) and were regenerated. Regenerated plants were subjected to marker excision analysis by cleaved amplified polymorphic sequences (CAPS). Furthermore, T_1_ plants were obtained from self-pollinating marker-free T_0_ plants containing W548L/S627I mutations in the *ALS* gene and were subjected to segregation analysis of the modified *ALS* gene and hyPBase expression vector, as well as analysis of transcript levels of the *ALS* gene and a BS-susceptibility test.
