# Supplementary material for: Precision genome editing in plants via gene targeting and piggyBac-mediated marker excision
Source: Plant J. 2014 Oct 6;81(1):160–8. doi: 10.1111/tpj.12693 (PMC4309413; doi:10.1111/tpj.12693)
Supplement: Supplementary file 2 — Figure S2. Molecular analysis of ALS GT-B1 and ALS GT-B1_hy T0 plants. [file tpj0081-0160-sd2.docx]

**
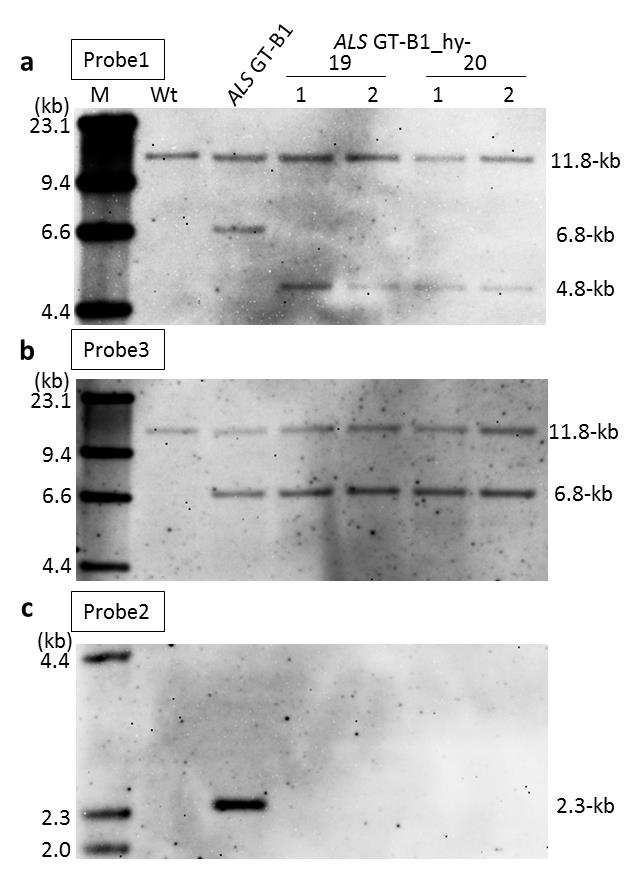
**

**Figure S2 Molecular analysis of ALS GT-B1 and ALS GT-B1_hy T_0_ plants.**

Southern blot analysis with probe1 (a), 2 (c) and 3 (b) shown in Figure 1a and 1b using *Mfe*I-digested genomic DNA of wild-type, *ALS* GT-B1 and GT-B1_hy T_0_ plants.
