# Supplementary material for: Precision genome editing in plants via gene targeting and piggyBac-mediated marker excision
Source: Plant J. 2014 Oct 6;81(1):160–8. doi: 10.1111/tpj.12693 (PMC4309413; doi:10.1111/tpj.12693)
Supplement: Supplementary file 3 — Figure S3. Strategy for the introduction of point mutations into Oscly1 locus via GT and subsequent marker excision from the GT locus using piggyBac transposon. [file tpj0081-0160-sd3.docx]

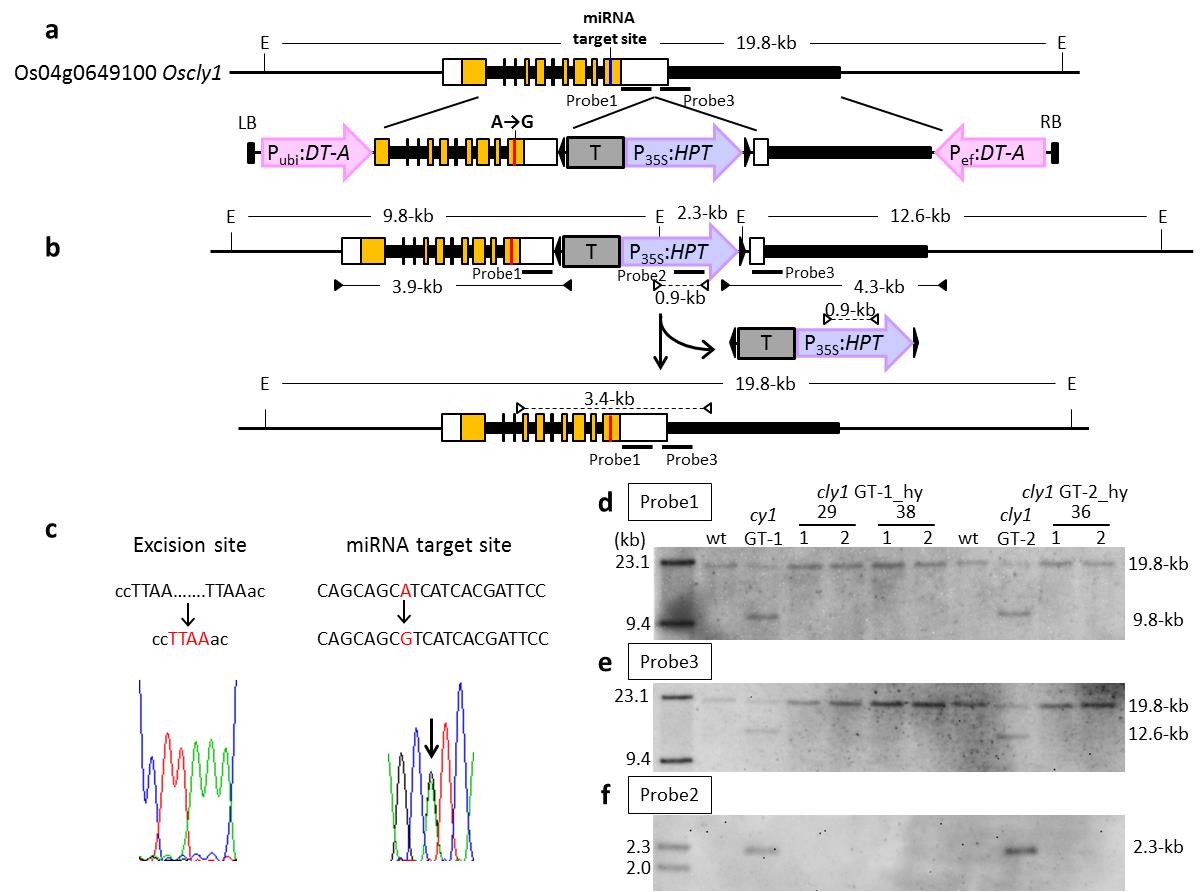


**Figure S3 Strategy for the introduction of point mutations into *Oscly1* locus via GT and subsequent marker excision from the GT locus using *piggyBac* transposon.**

(a) Schematic diagram of GT at the *Oscly1* (Os04g0649100) locus. The top line indicates the genomic structure of the wild-type *Oscly1* gene, which encodes a transcription factor containing a putative microRNA target site (blue line) 88-bp upstream of the stop codon. The bottom line shows the T-DNA region of the targeting vector carrying two *DT-A* gene expression cassettes as negative selection marker and a 6.0-kb fragment containing an *Oscly1* coding region (yellow boxes) with a single base substitution (CAGCAGCA/GTCATCACGATTCC, red lines) in the putative microRNA target site and *piggyBac* transposon (black triangle) harboring an *hpt* expression cassette as positive selection marker in the TTAA site of 3’-UTR (open boxes). LB, left border; RB, right border; E, *EcoR*V site. (b) Strategy for precise marker excision from the GT locus using *piggyBac* transposon. The top line reveals the structure of the modified *Oscly1* locus resulting from homologous recombination between the targeting vector and the wild-type locus. The bottom line represents the *Oscly1* locus modified by GT and subsequent precise marker excision via *piggyBac* transposition. The primer sets used for PCR to identify transgenic calli in which a GT event had occurred at *Oscly1* locus are shown as black arrows. White arrows indicate the primer sets used to evaluate the frequency of marker excision and re-integration via *piggyBac* transposition from the *Oscly1* locus. The numbers on each arrow reveal the length of the PCR fragments. Bars represent DNA probe fragments used for Southern blot analysis (d-f). (c) Sequencing chromatograms of the excision site and mutation site in T_0_ plants. Sequencing analysis revealed a single base substitution and precise marker excision in the *Oscly1* gene. (d-f) Southern blot analysis with probe1 (d), 2 (f) and 3 (e) shown in Fig. S3A and B using *EcoR*V-digested genomic DNA of wild-type, *Oscly1* GT-1, GT-2, *Oscly1* GT-1_hy and GT-2_hy T_0_ plants.
