# Supplementary material for: Precision genome editing in plants via gene targeting and piggyBac-mediated marker excision
Source: Plant J. 2014 Oct 6;81(1):160–8. doi: 10.1111/tpj.12693 (PMC4309413; doi:10.1111/tpj.12693)
Supplement: Supplementary file 4 — Figure S4. Herbicide bispyribac (BS)-tolerant phenotype of T1 calli. [file tpj0081-0160-sd4.docx]

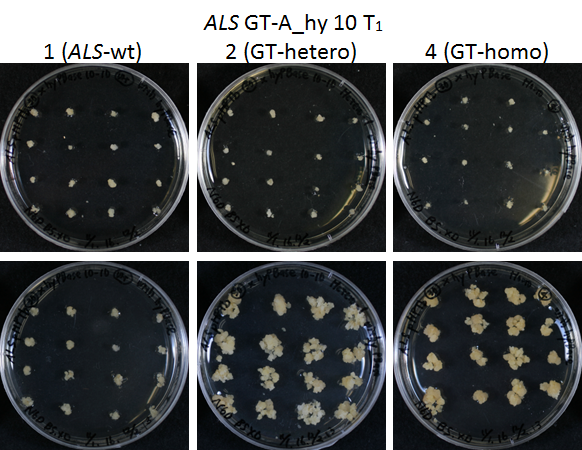


**Figure S4 Herbicide bispyribac (BS)-tolerant phenotype of T_1_ calli.**

GT line A_hy T_1_ calli at 0 (top) and 3 weeks (bottom) after the onset of BS selection. Calli carrying the modified *ALS* gene showed BS tolerance (ALS GT-A_hy 10-7 and 10-9).
