# Supplementary material for: Precision genome editing in plants via gene targeting and piggyBac-mediated marker excision
Source: Plant J. 2014 Oct 6;81(1):160–8. doi: 10.1111/tpj.12693 (PMC4309413; doi:10.1111/tpj.12693)
Supplement: Supplementary file 5 — Table S1. PCR analysis of piggyBac excision and re-integration events in ALS GT-B1_hy regenerated plants by hyPBase expression [file tpj0081-0160-sd5.docx]

**Table S1 PCR analysis of *piggyBac* excision and re-integration events in *ALS* GT-B1_hy regenerated plants by hyPBase expression**

| Line no. | No. of T_0_ plants analyzed | *piggyBac* excision from *OsALS* locus | | |  | Frequency of *piggyBac* excision (%) | | |
| --- | --- | --- | --- | --- | --- | --- | --- | --- |
|  |  | without marker | with marker | Total |  | without re-integration | with re-integration | Total |
| 5 | 20 | 17 | 1 | 18 |  | 85.0 | 5.6 | 90 |
| 9 | 20 | 20 | 0 | 20 |  | 100 | 0.0 | 100 |
| 11 | 20 | 20 | 0 | 20 |  | 100 | 0.0 | 100 |
| 12 | 20 | 17 | 0 | 17 |  | 85.0 | 0.0 | 85 |
| 19 | 20 | 16 | 0 | 16 |  | 80.0 | 0.0 | 80 |
| 20 | 20 | 20 | 0 | 20 |  | 100 | 0.0 | 100 |
| Ave. |  |  |  |  |  | 91.7 | 0.9 | 92.5 |
