# Supplementary material for: Precision genome editing in plants via gene targeting and piggyBac-mediated marker excision
Source: Plant J. 2014 Oct 6;81(1):160–8. doi: 10.1111/tpj.12693 (PMC4309413; doi:10.1111/tpj.12693)
Supplement: Supplementary file 6 — Table S2. Summary of GT experiments targeting the Oscly1 locus. [file tpj0081-0160-sd6.docx]

**Table S2 Summary of GT experiments targeting the *Oscly1* locus.**

| No. of *Agrobacterium* infected calli | No. of hygromycin- resistant calli | No. of targeted calli | No. of targeted calli with mutation* |
| --- | --- | --- | --- |
| 5,139 (25.6 g) | 74 | 4 | 4 |

*, Transgenic calli carrying an A/G mutation in the microRNA targeting site of *Oscly1* gene were identified by PCR analysis and sequencing with primer sets shown in Supplementary Fig. 3B. Two callus lines (*cly1* GT-1 and -2) were used for marker excision study (Table S3 and S4).
