# Supplementary material for: Precision genome editing in plants via gene targeting and piggyBac-mediated marker excision
Source: Plant J. 2014 Oct 6;81(1):160–8. doi: 10.1111/tpj.12693 (PMC4309413; doi:10.1111/tpj.12693)
Supplement: Supplementary file 7 — Table S3. PCR analysis of piggyBac excision and re-integration events in cly1 GT-1_hy plants regenerated by hyPBase expression [file tpj0081-0160-sd7.docx]

**Table S3 PCR analysis of *piggyBac* excision and re-integration events in *cly1* GT-1_hy plants regenerated by hyPBase expression**

| Line no. | No. of T_0_ plants analyzed | *piggyBac* excision from *Oscly1* locus | | |  | Frequency of *piggyBac* excision (%) | | |
| --- | --- | --- | --- | --- | --- | --- | --- | --- |
|  |  | without marker | with marker | Total |  | without re-integration | with re-integration | Total |
| 22 | 20 | 20 | 0 | 20 |  | 100 | 0 | 100 |
| 23 | 20 | 20 | 0 | 20 |  | 100 | 0 | 100 |
| 27 | 19 | 16 | 2 | 18 |  | 84.2 | 10.5 | 94.7 |
| 28 | 20 | 13 | 3 | 16 |  | 65.0 | 15.0 | 80.0 |
| 29 | 20 | 20 | 0 | 20 |  | 100 | 0 | 100 |
| 38 | 20 | 19 | 0 | 19 |  | 95.0 | 0 | 95.0 |
| Ave. |  |  |  |  |  | 90.7 | 4.3 | 95.0 |

*Cly1* GT-1 calli were infected with *Agrobacterium* harboring a hyPBase expression vector termed *cly1* GT-1_hy. About 20 regenerated plants from six independent *cly1* GT-1_hy were subjected to marker excision analysis by PCR analysis with the primer sets shown in Figure S3b.
