# Supplementary material for: Precision genome editing in plants via gene targeting and piggyBac-mediated marker excision
Source: Plant J. 2014 Oct 6;81(1):160–8. doi: 10.1111/tpj.12693 (PMC4309413; doi:10.1111/tpj.12693)
Supplement: Supplementary file 8 — Table S4. PCR analysis of piggyBac excision and re-integration events in cly1 GT-2_hy regenerated plants by hyPBase expression [file tpj0081-0160-sd8.docx]

**Table S4 PCR analysis of *piggyBac* excision and re-integration events in *cly1* GT-2_hy regenerated plants by hyPBase expression**

| Line no. | No. of T_0_ plants analyzed | *piggyBac* excision from *Oscly1* locus | | |  | Frequency of *piggyBac* excision (%) | | |
| --- | --- | --- | --- | --- | --- | --- | --- | --- |
|  |  | without marker | with marker | Total |  | without re-integration | with re-integration | Total |
| 21 | 22 | 22 | 0 | 22 |  | 100 | 0 | 100 |
| 33 | 21 | 21 | 0 | 21 |  | 100 | 0 | 100 |
| 36 | 25 | 25 | 0 | 25 |  | 100 | 0 | 100 |
| 37 | 25 | 24 | 0 | 24 |  | 96.0 | 0 | 96.0 |
| Ave. |  |  |  |  |  | 98.0 | 0.0 | 98.0 |

*Cly1* GT-2 calli were infected with *Agrobacterium* harboring a hyPBase expression vector termed *cly1* GT-2_hy. About 20 regenerated plants from four independent *cly1* GT-2_hy were subjected to marker excision analysis by PCR analysis with the primer sets shown in Figure S3b.
