# Supplementary material for: Precision genome editing in plants via gene targeting and piggyBac-mediated marker excision
Source: Plant J. 2014 Oct 6;81(1):160–8. doi: 10.1111/tpj.12693 (PMC4309413; doi:10.1111/tpj.12693)
Supplement: Supplementary file 9 — Table S4. PCR analysis of piggyBac excision and re-integration events in cly1 GT-2_hy regenerated plants by hyPBase expression [file tpj0081-0160-sd9.docx]

**Legends for Supporting Information**

**Figure S1** Experimental strategy for precise genome modification using the *piggyBac* transposon.

Four-week-old rice calli were inoculated with *Agrobacterium* harboring GT vector pKOD4/mALS and were selected for 4 weeks on medium containing 50 mg/l hygromycin B. Genomic DNA extracted from hygromycin-resistant calli was subjected to PCR analysis with the primer sets shown in **Figure 1b** to identify transgenic calli in which GT events had occurred at the *ALS* locus. GT callus lines were transferred to medium without the antibiotic meropenem, which kills *Agrobacterium*, and were cultured for 4 weeks. GT calli were again infected with *Agrobacterium* harboring an pPN/hyPBase expression vector (Nishizawa-Yokoi et al., 2014) encoding hyperactive *piggyBac* transposase (hyPBase) (Yusa et al., 2011) driven by the maize poly-ubiquitin gene 1 (Ubi-1) promoter. hyPBase transgenic calli were selected on medium containing geneticin (G418) and were regenerated. Regenerated plants were subjected to marker excision analysis by cleaved amplified polymorphic sequences (CAPS). Furthermore, T_1_ plants were obtained from self-pollinating marker-free T0 plants containing W548L/S627I mutations in the *ALS* gene and were subjected to segregation analysis of the modified *ALS* gene and hyPBase expression vector, as well as analysis of transcript levels of the *ALS* gene and a BS-susceptibility test.

**Figure S2** Molecular analysis of ALS GT-B1 and ALS GT-B1_hy T_0_ plants.

Southern blot analysis with probe1 (a), 2 (c) and 3 (b) shown in Figure 1a and 1b using *Mfe*I-digested genomic DNA of wild-type, *ALS* GT-B1 and GT-B1_hy T_0_ plants.

**Figure S3** Strategy for the introduction of point mutations into *Oscly1* locus via GT and subsequent marker excision from the GT locus using *piggyBac* transposon.

(a) Schematic diagram of GT at the *Oscly1* (Os04g0649100) locus. The top line indicates the genomic structure of the wild-type *Oscly1* gene, which encodes a transcription factor containing a putative microRNA target site (blue line) 88-bp upstream of the stop codon. The bottom line shows the T-DNA region of the targeting vector carrying two *DT-A* gene expression cassettes as negative selection marker and a 6.0-kb fragment containing an *Oscly1* coding region (yellow boxes) with a single base substitution (CAGCAGCA/GTCATCACGATTCC, red lines) in the putative microRNA target site and *piggyBac* transposon (black triangle) harboring an *hpt* expression cassette as positive selection marker in the TTAA site of 3’-UTR (open boxes). LB, left border; RB, right border; E, *EcoR*V site. (b) Strategy for precise marker excision from the GT locus using *piggyBac* transposon. The top line reveals the structure of the modified *Oscly1* locus resulting from homologous recombination between the targeting vector and the wild-type locus. The bottom line represents the *Oscly1* locus modified by GT and subsequent precise marker excision via *piggyBac* transposition. The primer sets used for PCR to identify transgenic calli in which a GT event had occurred at *Oscly1* locus are shown as black arrows. White arrows indicate the primer sets used to evaluate the frequency of marker excision and re-integration via *piggyBac* transposition from the *Oscly1* locus. The numbers on each arrow reveal the length of the PCR fragments. Bars represent DNA probe fragments used for Southern blot analysis (d-f). (c) Sequencing chromatograms of the excision site and mutation site in T_0_ plants. Sequencing analysis revealed a single base substitution and precise marker excision in the *Oscly1* gene. (d-f) Southern blot analysis with probe1 (d), 2 (f) and 3 (e) shown in Fig. S3A and B using *EcoR*V-digested genomic DNA of wild-type, *Oscly1* GT-1, GT-2, *Oscly1* GT-1_hy and GT-2_hy T_0_ plants.

**Figure S4** Herbicide bispyribac (BS)-tolerant phenotype of T_1_ calli.

GT line A_hy T_1_ calli at 0 (top) and 3 weeks (bottom) after the onset of BS selection. Calli carrying the modified *ALS* gene showed BS tolerance (ALS GT-A_hy 10-7 and 10-9).

**Table S1** PCR analysis of *piggyBac* excision and re-integration events in *ALS* GT-B1_hy regenerated plants by hyPBase expression

**Table S2** Summary of GT experiments targeting the *Oscly1* locus.

*, Transgenic calli carrying an A/G mutation in the microRNA targeting site of *Oscly1* gene were identified by PCR analysis and sequencing with primer sets shown in Supplementary Fig. 3B. Two callus lines (*cly1* GT-1 and -2) were used for marker excision study (Table S3 and S4).

**Table S3** PCR analysis of *piggyBac* excision and re-integration events in *cly1* GT-1_hy plants regenerated by hyPBase expression

*Cly1* GT-1 calli were infected with *Agrobacterium* harboring a hyPBase expression vector termed *cly1* GT-1_hy. About 20 regenerated plants from six independent *cly1* GT-1_hy were subjected to marker excision analysis by PCR analysis with the primer sets shown in Figure S3b.

**Table S4** PCR analysis of *piggyBac* excision and re-integration events in *cly1* GT-2_hy regenerated plants by hyPBase expression

*Cly1* GT-2 calli were infected with *Agrobacterium* harboring a hyPBase expression vector termed *cly1* GT-2_hy. About 20 regenerated plants from four independent *cly1* GT-2_hy were subjected to marker excision analysis by PCR analysis with the primer sets shown in Figure S3b.
